# Supplementary material for: Erk1/2-Dependent HNSCC Cell Susceptibility to Erastin-Induced Ferroptosis
Source: Cells. 2023 Jan 16;12(2):336. doi: 10.3390/cells12020336 (PMC9856753; doi:10.3390/cells12020336)
Supplement: Supplementary file 1 [file cells-12-00336-s001.zip › cells-2125561-supplementary.pdf]

Study population

Table S1. Study population. Characteristics of the 37 patients with head and neck cancer, who gave their written informed consent to spend a histological sample for this study, and the RNA and cDNA samples were of sufficient quality for further processing.

**sex**	male	36	97%
	female	1	3%
**age**	<=50	1	3%
	51–60	7	19%
	61–70	21	57%
	71–80	4	11%
	>80	4	11%
**ASA score**	ASA I/II	26	70%
	ASA III/IV	11	30%
**tumor site**	lips/oral cavity	4	11%
	oropharynx	16	43%
	hypopharynx	7	19%
	Larynx	10	27%
**histology**	squamous cell carcinoma	37	100%
**HPV/tumor localization**	P16-positive oropharynx SCC	9	56.25%
	P16-negative oropharynx SCC	7	43.75%
	P16-positive larynx SCC	1	10%
	P16-negative larynx SCC	9	90%
	P16-negative SCC of lips/oral cavity	4	100%
	P16-negative SCC of hypopharynx	7	100%
**UICC stage**	stage 1	4	11%
	stage 2	2	5%
	stage 3	11	30%
	stage 4a	17	46%
	stage 4b	2	5%
	stage 4c	1	3%
**HPV status**	negative (<70%)	27	73%
	positive (>=70%)	10	27%
**P53 status**	regular	11	30%
	irregular	26	70%
**treatment modality**	surgery	9	24%
	surgery & RT	7	19%
	surgery & RCHT/RIT	1	3%
	RCHT/RIT	18	49%
	RT	1	3%
	CHT	1	3%
